# Amylase Levels Are Useful for Diagnosing Omphalomesenteric Cysts: A Case Report

**DOI:** 10.3390/pediatric14010018

**Published:** 2022-03-09

**Authors:** Hiroko Yoshizawa, Keita Terui, Mitsuyuki Nakata, Tetsuya Mitsunaga, Shugo Komatsu, Takeshi Saito, Tomoro Hishiki

**Affiliations:** Department of Pediatric Surgery, Graduate School of Medicine, Chiba University, Chiba 260-8677, Japan; hyoshizawa1357@gmail.com (H.Y.); mitchinakachi@gmail.com (M.N.); tetsuya@z.email.ne.jp (T.M.); cbr@js5.so-net.ne.jp (S.K.); takksa5@gmail.com (T.S.); hishiki@chiba-u.jp (T.H.)

**Keywords:** amylase, diagnosis, omphalomesenteric cysts, remnants, hematoma

## Abstract

Omphalomesenteric cysts are an exceedingly rare type of embryologic remnant of the omphalomesenteric duct. Owing to its rarity and unspecific imaging findings, it is occasionally difficult to diagnose preoperatively. Herein, we report the case of a 15-month-old female with an omphalomesenteric cyst that presented as a painful abdominal mass. Imaging showed a 4 cm cystic lesion just beneath the umbilicus, which also contained a 1 cm enhanced lesion. On the immediate right side of this cyst, a 7 cm hematoma was found within the abdominal wall. Aspiration revealed bloody fluid with an amylase level of 38,250 U/L. She was then diagnosed with an omphalomesenteric cyst, with aberrant pancreas and associated hematoma of the abdominal wall. These findings were confirmed with laparotomy and subsequent pathological examinations. The high level of amylase in the cyst led us to speculate the existence of ectopic pancreatic tissue. Thus, amylase measurement may be considered for the diagnosis of an omphalomesenteric cyst.

## 1. Introduction

Omphalomesenteric cysts are an exceedingly rare type of embryologic remnant of the omphalomesenteric duct [1]. Owing to its rarity and unspecific imaging findings, it is occasionally difficult to diagnose preoperatively. We herein reported a rare case of omphalomesenteric cysts, in which the local level of amylase was useful for its diagnosis. 

## 2. Case Report

**Figure 1:**
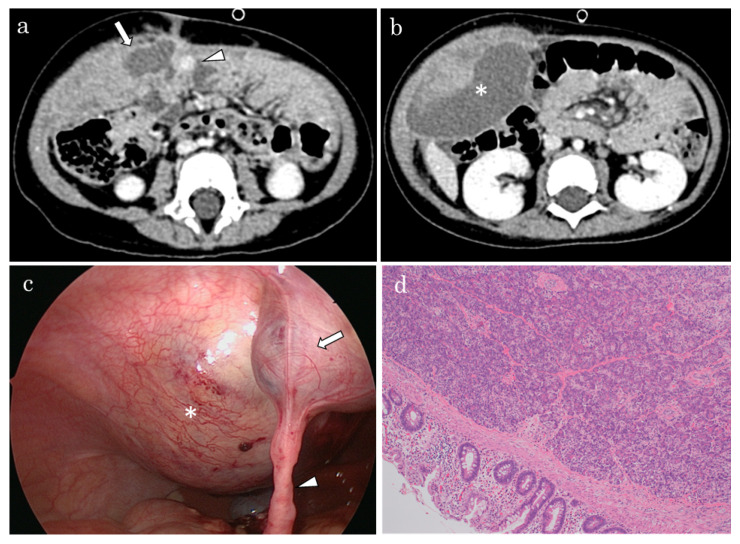
Contrast-enhanced computed tomography. (**a**) A 4 cm cystic lesion (arrow) located just beneath the umbilicus, containing an enhanced lesion of 1 cm in diameter (arrowhead). (**b**) On the immediate right side of this cyst, a 7 cm hematoma (*) is seen within the abdominal wall. (**c**) Laparoscopic observation reveals an omphalomesenteric cyst (arrow) beneath the umbilicus and a mesodiverticular band (arrowhead) connecting to the mesentery. These lesions are accompanied by a hematoma (*) extending to the right abdominal wall. (**d**) Pathological examination reveals that the omphalomesenteric cyst wall is lined with small intestinal and stomach mucosa, and the solid nodule contains pancreatic tissue mucosa.

A 15-month-old female presented with a painful abdominal mass. She had a history of ventricular septal defect, which was surgically treated at 8 months of age with a good postoperative course. Her vital signs were normal, except for a high temperature of 38.0 °C. Upon physical examination, an elastic hard mass was palpable on the right side of the abdomen, and the appearance of the umbilicus was normal. Blood examination indicated a white blood cell count of 14,500/μL, a C-reactive protein level of 2.4 mg/dL, a hemoglobin level of 9.6 g/dL, and an amylase level of 98 U/L (44−132 U/L). Abdominal radiography revealed no signs of intestinal obstruction. Abdominal ultrasonography (US) revealed a large intraabdominal cyst, but contrast-enhanced computed tomography (CT) showed that a cystic lesion that was 4 cm in diameter was located in the abdominal wall, beneath the umbilicus. The cystic lesion contained a 1 cm enhanced lesion (Figure 1a). On the immediate right side of this cyst, a 7 cm hematoma was found within the abdominal wall (Figure 1b). Aspiration revealed bloody fluid with an amylase level of 38,250 U/L, and no bacterial infection was observed. Based on imaging findings and the high amylase level, she was diagnosed with an omphalomesenteric cyst, with aberrant pancreas and associated hematoma of the abdominal wall. Laparoscopy further revealed a mesodiverticular band connecting to the mesentery, in addition to the cyst. Moreover, the omphalomesenteric cyst was connected to the hematoma, which extended to the right abdominal wall (Figure 1c). During surgery, the mesodiverticular band was detached laparoscopically, the omphalomesenteric cyst was completely removed via circumumbilical and small median incisions, and the hematoma was excised to the greatest extent possible. Pathological examination revealed that the omphalomesenteric cyst wall was lined with small intestinal and stomach mucosa, and pancreatic tissue mucosa was found in the solid nodule (Figure 1d). The CT-enhanced lesion was also confirmed as an ectopic pancreatic tissue. The hematoma wall was not lined with an epithelial structure, and no malignant findings were observed. The patient was discharged after 1 week postoperatively, and there were no problems during the 8 years of observation. 

## 3. Discussion

The omphalomesenteric duct is an embryonic structure connecting the yolk sac and the midgut, which normally disappears at approximately 5–9 weeks of gestation. The most common type of remnant, the Meckel’s diverticulum, accounts for 82–96% of all types and is found in 2% of autopsies [1]. In contrast, omphalomesenteric cysts are an exceedingly rare type, accounting for 1% of all omphalomesenteric duct remnant cases [1,2]. Omphalomesenteric cysts are characterized by cystic structures that are located in the body wall or midportion of the vitelline duct and are connected to the ileum and body wall by fibrous bands [1]. 

The symptoms of omphalomesenteric remnants depend on their location. If the remnant opens or is located in the urachus, urachal effusion, bleeding, and/or inflammation are usually observed. If it communicates with the intestine, abdominal pain and/or gastrointestinal bleeding are the main symptoms [1]. However, if an omphalomesenteric cyst has a closed cavity, patients can be asymptomatic before progression. In the present case, the secretion of pancreatic enzymes from ectopic pancreatic tissue might have caused the development of hematoma and the omphalomesenteric cyst, presumably resulting in abdominal pain. Such situations make diagnosis difficult, especially in cases that are localized to the abdominal wall with a normal-looking umbilicus. 

Differential diagnosis of pediatric body wall masses includes rectus abdominis hernia; urachus remnants; rectus abdominis hematoma; abdominal wall abscess; hematoma; lymphatic/vascular malformations; and malignant tumors, such as rhabdomyosarcoma and other rare sarcomas. Although diagnosis is important to exclude malignancies, imaging findings of omphalomesenteric cysts are often inconclusive due to their non-specific appearance, as was observed in our case despite the use of other modalities, including US and magnetic resonance imaging (not shown).

In this case, where malignancies could not be eliminated, a high amylase level of the content fluid was helpful in the diagnosis of ectopic pancreatic tissue, since it is well-known that ectopic pancreatic tissue may be contained in omphalomesenteric remnants. To the best of our knowledge, there have been no reports on the use of amylase levels for the preoperative diagnosis of omphalomesenteric remnants. 

Over 70% of the ectopic pancreatic tissue is located in the gastrointestinal tract, mainly in the duodenum and colon [3]. Moreover, ectopic pancreatic tissue in the duodenum was found in 13.4% of autopsy materials [3]. Interestingly, omphalomesenteric remnants are also reported to frequently contain ectopic pancreatic tissue [1]. In Meckel’s diverticulum, the frequency of ectopic tissue has been well studied (gastric mucosa (62%), pancreatic tissue (6%), combined gastric mucosa and pancreatic tissue (5%), and other tissues (16%)) [4]. However, the frequency of ectopic tissue in omphalomesenteric cysts is unclear due to their rarity. According to an existing case report of an omphalomesenteric cyst, pancreatic tissue was involved in six cases (Table 1) [5,6,7,8,9,10]. The age of the patients ranged from 3 months to 2 years, and four patients were male. Various non-specific symptoms in the umbilicus were observed, including abrasion [7], tumor [6], swelling after trauma [5], erythema [9,10], discharge [8,9], and oozing [7]. The lesions were either cysts [5,6,7,9] or ducts [8,10]. There was only one case of an accompanying hematoma, as similarly observed in the present case [5]. No patient underwent aspiration, or preoperative measurement of their serum or local amylase levels. Among them, only one patient was preoperatively diagnosed with an omphalomesenteric cyst based on imaging findings [9]. Furthermore, all patients underwent surgical resection and had good postoperative courses. Therefore, the present case was the first case of an omphalomesenteric cyst that was diagnosed with high amylase levels upon aspiration of the hematoma fluid.

## 4. Conclusions

Apart from imaging findings, a high local level of amylase may indicate the existence of ectopic pancreatic tissue, which is helpful for the diagnosis of omphalomesenteric cysts. 

## Figures and Tables

**Table 1:** Summary of cases of omphalomesenteric cyst with the ectopic pancreatic tissue. Age at operation, sex, umbilical symptom, and the presence of stomach and intestinal tissues in pathological diagnosis were compared between the present case and previous reports.

No.	Age/Sex	Umbilical Abnormality	Stomach Tissue	Intestinal Tissue	Reference
1	1 year/M	Swelling after trauma	No	Yes	[5]
2	2 years/M	Umbilical tumor	No	No	[6]
3	2 years/M	Abrasion, oozing	Yes	Yes	[7]
4	3 months/F	Umbilical discharge	No	Yes	[8]
5	16 months/M	Umbilical discharge, erythema	No	No	[9]
6	9 months/F	Erythema	No	Yes	[10]
7	15 months/F	None	Yes	Yes	Present case

## Data Availability

Data sharing not applicable. No new data were created or analyzed in this study. Data sharing is not applicable to this article.
